# Relevance of Quality of Life Assessment for Multiple Sclerosis Patients with Memory Impairment

**DOI:** 10.1371/journal.pone.0050056

**Published:** 2012-12-11

**Authors:** Karine Baumstarck, Françoise Reuter, Mohamed Boucekine, Valérie Aghababian, Irina Klemina, Anderson Loundou, Jean Pelletier, Pascal Auquier

**Affiliations:** 1 Department of Public Health, EA3279 Self-perceived Health Assessment Research Unit, Timone University, APHM, Marseille, France; 2 Departments of Neurology and CRMBM CNRS6612, Timone University Hospital, APHM, Marseille, France; 3 Department of Psychology, EA 3273 Psychology of Cognition, Language, and Emotion Research Centre, Aix-Marseille University, France; University of Missouri-Kansas City, United States of America

## Abstract

**Background:**

Memory disturbances, in particular episodic verbal memory dysfunction, are the most frequent cognitive impairment observed in multiple sclerosis (MS) patients. The use of self-reported outcomes for evaluating treatment and managing care of these subjects has been questioned. The aim of this study was to provide new evidence about the suitability of self-reported outcomes for use in this impaired population by exploring the internal structure, reliability and external validity of a specific quality of life (QoL) instrument, the Multiple Sclerosis International Quality of Life questionnaire (MusiQoL).

**Methods:**

*Design*: cross-sectional study. *Inclusion criteria*: MS patients of any disease subtype. *Data collection*: sociodemographic (age, gender, marital status, education level, and occupational activity) and clinical data (MS subtype, Expanded Disability Status Scale, disease duration); QoL (MusiQoL and SF36); and memory performance (Grober and Buschke test). In accordance with the French norms of the memory test, non-impaired and impaired populations were defined for short- and long-delay free composites and for short- and long-delay total composites. For the 8 populations, psychometric properties were compared to those reported from the reference population assessed in the validation study.

**Principal Findings:**

One hundred and twenty-four consecutive patients were enrolled. The analysis performed in the impaired populations showed that the questionnaire structure adequately matched the initial structure of the MusiQoL. The unidimensionality of the dimensions was preserved, and the internal/external validity indices were close to those of the reference population.

**Conclusions/Significance:**

Our study suggests that memory dysfunction did not compromise the reliability or validity of the self-reported QoL questionnaires.

## Introduction

The use of self-reported outcomes in subjects with cognitive dysfunction is of particular concern [1]. The main argument against using self-reported quality of life (QoL) information from patients with cognitive dysfunction is based on the fact that QoL instruments were not developed for these specific individuals. Although there is little evidence concerning the reliability and validity of health status measures in cognitively impaired patients [1], two perspectives have been reported. While some authors argue that cognitively impaired individuals are unable to produce valid QoL measures [2], [3], others reported empirical evidence suggesting that individuals with a moderate degree of cognitive impairment can perform reliable QoL assessments [4], [5], [6], [7]. Most of the studies collected information from patients with severe mental disorders [8], [9], [10] or older populations [11] presenting with dementia or other severe cognitive impairment [11], [12], [13].

While cognitive impairment occurs in approximately 50% of MS patients [2], [14], even during the early stages of the disease [15], the extent to which MS patients with cognitive dysfunction can validly self-report their QoL is a crucial issue that has not been sufficiently examined. To our knowledge, only two major studies reported data from MS patients [5], [6], and both suggested that cognitive decline does not compromise the reliability/validity of self-reported health measures. However, these studies did not report how the structure described in the impaired samples fit with the initial structure of the tested instrument, and they provided restricted data regarding validity and reliability. We previously reported data providing strong arguments to support the conclusion that MS patients with executive dysfunction, as determined by the Stroop test, are reliable and consistent when answering the MusiQoL questionnaire [7]. These robust results needed to be confirmed by assessing other cognitive functions, such as memory. Memory disturbances, especially episodic verbal memory dysfunction, appear to be the most frequently affected cognitive function in MS [16], [17]. To provide new evidence about the suitability for using self-reported QoL information in this impaired population, we explored the internal structure, reliability and external validity of a specific QoL instrument, the Multiple Sclerosis International Quality of Life questionnaire (MusiQoL) [18]. The study sample included MS subjects with or without short- or long-delay verbal memory impairment.

## Methodology

This study relied on a cross-sectional design and was performed in the neurology department of a public French academic teaching hospital (Marseille, France). The inclusion criteria were as follows: a MS diagnosis according to the McDonald criteria [19], age ≥18 years, any subtype of MS, outpatient status, no neurological disease (other than MS), no history of severe mental illness (except depression disorder), no dementia (Mini Mental State Examination score <24), no history of alcohol/drug abuse, and native French speaking. The French Ethics Committee approved the study (Comité Consultatif de Protection des Personnes dans la Recherche Biomédicale, Marseille 2, France) and patients gave their written consent to participate. Sociodemographic (age, gender, marital status, education level, and occupational activity) and clinical (MS subtype and disease duration) data were recorded for each patient. MS disability was assessed using the Expanded Disability Status Scale (EDSS).

QoL was assessed by means of the MusiQoL, which is a well-validated questionnaire that describes the following nine dimensions and yields a global index score: activity of daily living (ADL), psychological well-being (PWB), symptoms (SPT), relationships with friends (RFr), relationships with family (RFa), relationships with health care system (RHCS), sentimental and sexual life (SSL), coping (COP), and rejection (REJ). The indicators of the reference population [18] are listed in table 1. QoL assessment was performed using the Short Form 36 (SF36), which is a generic questionnaire [20] describing eight subscales: physical function (PF), social function (SF), role physical (RP), role emotional (RE), mental health (MH), vitality (V), bodily pain (BP), and general health (GH). Two composite scores (physical and mental, PCS-SF36 and MCS-SF36) were also calculated.

**Table 1:** Internal structural validity/reliability/unidimensionality of the reference population*.

	M±SD	IIC^1^ Min-Max	IDV^2^ Min-Max	Floor%	Ceiling %	Alpha^3^	INFIT^4^	Missing values %
Activity of daily living	54,19±27,09	0,66-0,81	0,02-0,49	1,3	4,6	0,92	0,86-1,2	1,4
Psychological well-being	55,92±23,8	0,67-0,76	0,09-0,41	2,4	4,6	0,85	0,81-1,13	0,9
Relationships with friends	63,67±25,54	0,69-0,78	0,04-0,36	2,4	13	0,75	0,84-1,15	7,4
Symptoms	66,68±23,44	0,48-0,65	0,06-0,41	0,7	10,3	0,80	0,75-1,17	0,7
Relationships with family	75,21±23,07	0,62-0,68	0,04-0,38	0,8	25,7	0,86	0,88-1,07	2,3
Relationships with health care system	77,80±20,17	0,42-0,56	0,05-0,32	0,3	24,5	0,68	0,83-1,18	2,6
Sentimental and sexual life	61,06±31,86	0,75-0,75	0,15-0,43	7,6	18,7	0,85	0,98-1	18,8
Coping	62,45±30,62	0,66-0,66	0,12-0,45	5,8	21,1	0,80	0,97-1	5,1
Rejection	75,43±26,02	0,60-0,60	0,13-0,41	1,5	32,9	0,74	0,95-1,04	9
Index	65,85±14,75	0,66-0,81	0,02-0,49	1,3	4,6	0,92	0,86-1,2	1,4

1 Item-Internal Consistency,

2 Item Discriminant Validity,

3 Cronbach's alpha,

4 Rasch statistics.

* Simeoni M, Auquier P, Fernandez O, Flachenecker P, Stecchi S, Constantinescu C, Idiman E, Boyko A, Beiske A, Vollmer T, Triantafyllou N, O'Connor P, Barak Y, Biermann L, Cristiano E, Atweh S, Patrick D, Robitail S, Ammoury N, Beresniak A, Pelletier J (2008) Validation of the Multiple Sclerosis International Quality of Life questionnaire. Mult Scler 14:219–230.

Episodic verbal memory performance was explored using the French version of a free/cued recall test designed by Grober and Buschke [21]. The test was administered in a standardised manner by the same senior psychologist (FR), who was intensively trained in test administration. The same instructions were given to the subjects prior to each trial. The test used a 16-word list [http://www.neuropsycho.ulg.ac.be/], and the words were grouped into four semantic categories with four words each. The subject was asked to recall as many words as possible during three consecutive trials: 3 short-delay free recalls and 3 short-delay cued recalls. For each trial, the sum of the 3 short-delay free and 3 short-delay cued recalls is computed to yield three short-delay total recalls. Later, a fourth trial is performed consisting of one long-delay free and one long-delay cued recall. The sum of the long-delay free and long-delay cued recalls constitute the long-delay total recall.

For each subtest, the subject was defined as impaired or non-impaired by applying French normative values according to age, gender, and education level [21] A patient was considered to be cognitively impaired if the short-delay and long-delay subtest total score was lower than the fifth percentile and if the short-delay and long-delay free subtest z-score was lower than −1.65 [21]. Patients were categorized into the following eight groups:

1. Short-delay free memory: non-impaired (three normal performances on the short-delay free recalls) and impaired (one or more abnormal performance) populations;
2. Short-delay total memory: non-impaired (three normal performances of the short-delay total recalls) and impaired (one or more abnormal performance) populations;
3. Long-delay free memory: non-impaired (normal performance of long-delay free recall) and impaired (abnormal performance) populations;
4. Long-delay total memory: non-impaired (normal performance of long-delay total recall) and impaired (abnormal performance) populations.

### Statistical analysis

Statistical analyses were performed on the eight populations defined above using the same procedure reported in the initial validation publication (reference population). For each group, psychometric properties were compared to those reported from the reference population assessed in the validation study [18], [22], [23]. The definitions of the main psychometric properties are summarized in table S1.

The multidimensional structure (construct validity) was verified using the multi-trait/multi-item analysis programme [24]. Internal structural validity was assessed by investigating item-dimension correlations. Item internal consistency (IIC) was calculated by correlating each item with its scale, and item discriminant validity (IDV) was assessed by determining the extent to which items correlated with the dimension they were hypothesised to represent, compared to correlations with other dimensions. Floor and ceiling effects were reported to assess the homogeneous repartition of the response distribution. For each dimension, internal consistency reliability was evaluated by Cronbach's alpha coefficient [25].

The unidimensionality of each dimension was explored by computing item goodness-of-fit statistics (INFIT) issued from Rasch analyses [26]. INFIT values ranging from 0.7 to 1.2 ensure that all scale items tend to measure the same concept.

To explore external validity, Spearman's correlation coefficients were used to investigate relationships between MusiQoL and SF36 dimensions in each group, and the associations between MusiQoL dimension scores and sociodemographic and clinical features were reported as in the validation study. For qualitative variables, mean dimension scores of the MusiQoL were compared across patient groups (e.g., gender, educational level, marital status, and occupational status) using one-way analysis of variance. Quantitative variables (e.g., age, EDSS score, and MS duration) were analysed using Spearman's correlation coefficients. Acceptability was assessed by calculating the percentage of missing data per dimension. Data analyses were performed using SPSS 11.0, MAP-R, and WINSTEP software.

### Suitability indices

We provided suitability indices to quantify how each of the 8 structures matched with the initial structure (reference structure, see appendix) of the questionnaire. Decision rules were used to define satisfactory properties according to appropriate standards [22], [23]. These rules were established by three experts in QoL (KB, AL, and PA). All the decision rules are detailed in table 2. The means of different proportions were calculated to produce two suitability indices: the suitability index of the ‘construct validity’ and the suitability index of the ‘external validity’.

**Table 2:** The suitability indices: decision rules for the construct validity and the external validity.

Indices of validity	Result
**1. Regarding the construct validity/internal structure**	
Proportion of dimensions with IIC non-exceeded 0.2 from the reference	0–100%*
Proportion of dimensions with IDV non-exceeded 0.2 from the reference	0–100%*
Proportion of dimensions with items correlated higher with the dimension they were hypothesized to represent as compared to correlations with other dimensions	0–100%*
Proportion of dimensions with a Cronbach's alpha coefficient of at least 0.70 excepted for the relationships with RHCS for which a coefficient of at least 0.68 (reference's coefficient):	0–100%*
Proportion of dimensions with INFIT ranged between 0.7 and 1.3	0–100%*
Proportion of dimensions with percentages of missing values non exceeded 10% from the reference	0–100%*
Proportion of dimensions with floor effect non exceeded 10% from the reference	0–100%*
Proportion of dimensions with ceiling effect non exceeded 10% from the reference	0–100%*
Suitability index of ‘construct validity’	Mean of the proportions
**2. Regarding the external validity**	
MusiQoL and SF36:Proportion of dimensions which met the following conditions issued from the initial validation study: i) correlation coefficient between ADL and PF or RP or V higher than 0.50 and stronger than the other correlations; ii) correlation coefficient between PWB and MH higher than 0.50 and stronger than the other correlations; iii) all other correlation coefficients inferior to 0.40.	0–100%*
MusiQoL and age:Proportion of dimensions with a correlation coefficient <0.40.	0–100%*
MusiQoL and MS duration:Proportion of dimensions with a correlation coefficient <0.40	0–100%*
MusiQoL and EDSS:Proportion of dimensions which met the following conditions: i) correlation coefficient between ADL and EDSS >0.4 and stronger than the other correlations; ii) all other correlation coefficients inferior to 0.40.	0–100%*
MusiQoL and gender, educational level, marital status, and occupational status:Proportion of dimensions with effect size non-exceeded 0.2 from the reference	0–100%*
Suitability index of ‘external validity’	Mean of the proportions

* 100% when the 9 dimensions met the condition, 89% when 8 dimensions met the condition, etc…;

## Results

One hundred and twenty-four consecutive patients were enrolled. The mean age was 45 years (SD 11), 71 (57,3%) of the patients were women, and 58 (47,2%) had more than 12 years of education. MS subtypes included 61 (49,2%) secondary progressive, 36 (29,0%) relapsing-remitting, 20 (16,1%) primary progressive, and 7 (5,6%) clinically isolated syndromes. The EDSS median was 4.75 (minimum 1.00, maximum 8.00) and the median disease duration was 9.86 years (minimum 0, maximum 31). From the French normative values [27], the number of subjects for each of the analysed populations were: 1) short-delay free populations: 74 non-impaired and 38 impaired individuals; 2) short-delay total populations: 101 non-impaired and 21 impaired individuals; 3) long-delay free populations: 66 non-impaired and 45 impaired individuals; 4) long-delay total populations: 102 non-impaired and 19 impaired individuals. The mean dimension scores and indices for each non-impaired and impaired population are reported in tables S2, S3, S4, and S5. Missing values were more frequent in the impaired populations compared to the non-impaired populations but never exceeded 10% (except for the SSL dimension in the impaired long-delay total group).

### Construct validity

The proportions of dimensions with IIC that were greater than 0.2 from the reference were higher in the impaired populations than the non-impaired populations, with lower suitability indices for the non-impaired short-delay free population (61,1%). In contrast, the proportions of dimensions with IDV that were not greater than 0.2 from the reference were more satisfactory in the non-impaired populations, with lower suitability indices for the impaired short-delay free population (38,9%) (table S2).

The correlation for each item with its contributive dimension was higher than with the others (IDV) for 7 or 8 dimensions in the 4 non-impaired populations but only 2, 4 or 6 dimensions in the impaired long-delay total, short-delay total, and short-delay free populations, respectively. Cronbach's alpha coefficients were satisfactory in at least 8 of 9 dimensions in the four impaired populations. The non-impaired populations showed satisfactory INFIT statistics more frequently than the 4 impaired populations, except for the two short-delay free populations, which had 72% of the dimensions with INFIT inside the range in the non-impaired population versus 83% in the impaired population (tables S2, S3, S4, and S5). Floor effects were less than 10% from the reference for 8 or 9 dimensions, regardless of the population. The proportion of dimensions with a ceiling effect exceeded 10% from the reference were higher or equal in the impaired populations (range: 44,4 to 77,8%) compared to the non-impaired populations (range: 55,6 to 77,8%). The details are provided in tables S2, S3, S4, and S5.

**Figure 1:**
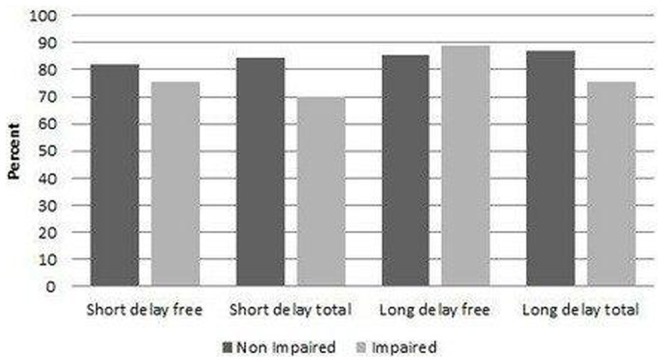
Suitability indices of construct validity.

The suitability indices of construct validity in the impaired populations were ranged from 71% (the impaired short-delay total population) to 89% (the impaired long-delay free population). These data are summarised in table 3 and figure 1.

**Table 3:** Suitability indices of construct validity and external validity.

	Short delay free		Short delay total		Long delay free		Long delay total	
Construct validity	NI 74	I 38	NI 101	I 21	NI 66	I 45	NI 102	I19
% of dimensions with IIC non exceeded 0.2 from ref	61,1	94,4	77,8	88,9	72,2	94,4	72,2	83,3
% of dimensions with IDV non exceeded 0.2 from ref	77,8	38,9	72,2	44,4	100	100	100	77,8
% of dimensions with IDV<IIC	77,8	66,7	88,9	44,4	77,8	77,8	88,9	22,2
% of dimensions with Cronbach's alpha coefficients > = 0.7 or > = ref	88,9	88,9	88,9	88,9	66,7	100	88,9	88,9
% of dimensions with INFIT ranged [0.7–1.3]	72,2	83,3	94,4	72,2	88,9	83,3	88,9	66,7
% of dimensions with MV <10% from ref	100	88,9	100	88,9	100	88,9	88,9	100
% of dimensions with Floor <10% from ref	100	88,9	88,9	88,9	100	88,9	88,9	100
% of dimensions with Ceiling <10% from ref	77,8	55,6	66,7	44,4	77,8	77,8	77,8	66,7
Total	82,0	75,7	84,7	70,1	85,4	88,9	86,8	75,7

External validity	NI 74	I 38	NI 101	I 21	NI 66	I 45	NI 102	I19
SF36: % of dimensions meeting three conditions*	88,9	77,8	88,9	55,6	88,9	88,9	77,8	55,6
Age: % of dimensions with correlation coefficient <0.40	100	100	100	100	100	100	100	88,9
EDSS: % of dimensions meeting two conditions**	88,9	88,9	100	88,9	100	88,9	88,9	77,8
MS duration: % of dimensions with correlation coefficient <0.40	100	100	100	100	100	100	100	100
Gender: % of dimensions with ES <0.2 from ref	88,9	66,7	100	44,4	100	55,7	100	44,4
Educational level: % of dimensions with ES <0.2 from ref	88,9	33,3	66,7	66,7	77,8	22,2	55,6	55,6
Marital status: % of dimensions with ES <0.2 from ref	100	100	100	88,9	88,9	100	100	88,9
Occupational status: % of dimensions with ES <0.2 from ref	88,9	55,6	88,9	33,3	77,8	22,2	77,8	55,6
Total	93,1	77,8	93,1	72,2	91,75	72,2	87,5	70,9

NI non-impaired, I impaired.

* MusiQoL and SF36: the three conditions were: i) correlation coefficient between ADL and PF or RP or V higher than 0.50 and stronger than the other correlations; ii) correlation coefficient between PWB and MH higher than 0.50 and stronger than the other correlations; iii) all other correlation coefficients inferior to 0.40.

** MusiQoL and EDSS: the two conditions were: i) correlation coefficient between ADL and EDSS>0.4 and stronger than the other correlations; ii) all other correlation coefficients inferior to 0.40. The score was 100% when all the dimensions met the condition.

### External validity

The results are summarised in the table 3. The expected level of correlation between the ADL dimension of the MusiQoL and the ‘physical-like’ dimensions of the SF36 was never found in the impaired populations (the correlation coefficient was always less than 0.50) and was always found across all non-impaired populations. As expected, the mental health dimension of the SF36 was mainly statistically associated with the psychological well-being dimension of the MusiQoL across all analysed populations except the impaired long-delay total population (correlation coefficient 0.39). In the impaired populations, all other relationships between the MusiQoL and the SF36 dimensions had a correlation coefficient <0.40, except for seven of them (examples: REJ and SF found in two impaired populations, SPT and RE in two other impaired populations). The suitability indices ranged from 56 to 89% in the impaired populations.

Age and disease duration almost never correlated with MusiQoL dimensions; age correlated with RHCS in the impaired long-delay total population. The correlation between the EDSS and ADL dimension was higher than 0.40 in five populations (3 non impaired and two impaired populations), and two of them had a 100% suitability index. A low suitability index was found for the impaired long-delay total population due to an unexpectedly high correlation between the EDSS and REJ dimension.

The proportions of dimensions with effect size less than 0.2 from the reference for gender were satisfactory for the non-impaired populations, with suitability indices ranging from 88,9 to 100%. This contrasted with the impaired populations, which had lower indices, ranging from 44,4 to 66,7%. The suitability indices regarding the decision rule of the relationship between MusiQoL and marital status were high for both the impaired and non-impaired populations. The suitability indices were less satisfactory for educational level and occupational status, which were always worse in the impaired populations.

**Figure 2:**
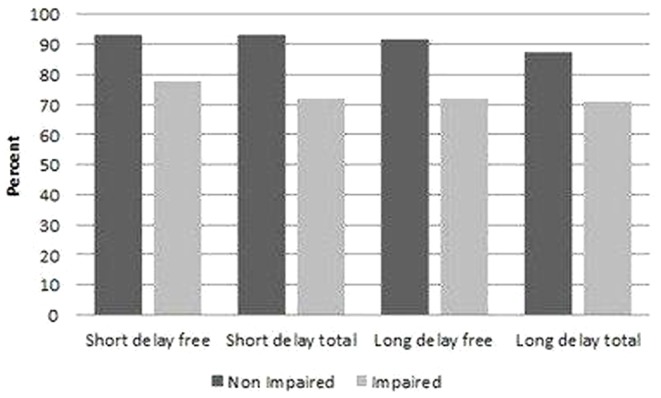
Suitability indices of external validity.

In summary, the suitability indices of external validity in the non-impaired populations ranged from 71% to 78%. The suitability index was higher in the impaired short/long-delay free population compared to the impaired short/long-delay total population. These data are summarised in table 3 and figure 2.

## Discussion

The assessment of quality of life has received increasing recognition as an outcome parameter in MS research, but whether self-reported information is reliable in cognitively impaired patients and to what extent QoL measurement remains valid in this context are major considerations. Therefore, it is necessary to determine if the initial internal structures of the self-reported measures are well adapted when QoL measures are used for cognitively impaired individuals and to confirm if psychometric properties are satisfactory in these populations [1].

As reported in previous studies [6], [7], [28], our results provide strong evidence to support the conclusion that cognitively impaired MS patients, as defined by memory dysfunction, reliably and consistently answer the MusiQoL questionnaire.

Regarding construct validity, the indicators of the impaired populations can be considered satisfactory compared to the reference population. The unidimensionality of each of these dimensions was supported by the satisfactory INFIT statistics. While IIC values reported in the impaired populations were closer to those of the reference population than the non-impaired populations, the proportions of dimensions with IDV values superior to the IIC values were more satisfactory in the non-impaired populations. Internal consistency coefficients were similar to those of the initial reference population despite patients' cognitive status, with the exception of the relationship with health care in two impaired populations (impaired short and long-delay total populations) and coping in three non-impaired populations (non-impaired short-delay free, short-delay total and long-delay free populations). Floor effects were similar to those reported in the initial validation publication, and ceiling effects were slightly different than those reported in the initial validation publication, mainly in the impaired populations.

Concerning external validity, the impaired populations provided satisfactory suitability indices. The MusiQoL scores of these populations were quite consistent with those of the SF36 compared to the reference population. As expected, psychological well-being was highly correlated to the mental health dimension of SF36. However, activity of daily living was not linked to the ‘physical-like’ dimensions of SF36 in the impaired populations. These findings support the validity of the MusiQoL adding information not covered by generic questionnaires [29].

Regarding the links between MusiQoL scores and age, handicap score (EDSS), disease duration, and marital status, they were closer to the initial reference population regardless of cognitive status in impaired and non-impaired populations. However, links between MusiQoL scores and gender, educational level, and occupational status were less satisfactory in the impaired populations.

In summary, the suitability indices of the impaired populations were lower compared to those of the non-impaired populations (except for the long-delay free populations), but they could be considered totally acceptable considering the small sample size of some of the defined populations. The suitability indices of impaired short-term memory seemed less satisfactory than the impaired long-term memory, suggesting that verbal memory impairment among MS subjects is less a consequence of a function of impaired retrieval than inadequate initial learning. This is in line with previous studies showing that the primary memory problem can be the initial learning of information [30], [31].

Patients with MS seemed to require more repetitions to reach a predetermined learning criterion, but once that information has been acquired, recall and recognition are at the same level as for healthy controls [30], [31]. However, long-term memory that refers to the ability to learn new information and to recall that information at a later time point is a consistently impaired cognitive function in MS patients [17], [30].

There are several strengths and limitations of this study:

1. The sample size was small but similar to other studies [5], [6] and explained some unsatisfactory properties in impaired populations. Despite this limitation, the cumulative argument of the present study should ensure the relevance of self-reported quality of life assessments for patients with cognitive disorders.
2. The representativeness of our sample should also be discussed. Our patients had a more severe disability profile and a higher proportion of secondary progressive disease compared to international and European MS populations [18], [32]. Data on exacerbation status was not collected. Nevertheless, the present study did not focus on the most severe cases because patients with dementia or those unable to be assessed using neuropsychological tests were not included. However, the proportion of cognitively impaired subjects, from 15,7% (impaired long-delay total) to 40,5% (impaired long-delay free), was in accordance with the literature [2], [14], [30] and was comparable to other studies with similar objectives [5], [6]. Future studies should be performed to confirm these findings in different subtypes of MS.
3. The proportions of immediate memory dysfunction were higher than the total memory dysfunction. This is in line with previous studies demonstrating that the primary memory problem might be the initial learning of information [30], [31]. Long-term memory that refers to the ability to learn new information and recall it at a later time point is one consistently impaired cognitive function in MS [17], [30]. Currently, there is consensus that multiple aspects of memory are impaired, including attention-mediated or executive aspects, such as encoding and retrieval, and perhaps even consolidation and recognition in some circumstances [33]. Nevertheless, we are conscious of the fact that the impaired and non-impaired populations of the present study partially overlapped.
4. Another important aspect of this study concerns our definition of cognitive dysfunction. Indeed, cognition can be defined as the mental process of knowing, including awareness, perception, reasoning, and judgment. We studied composite memory function because it is among the most commonly affected cognitive domains in MS patients [16], [34]. Verbal memory seems to be less commonly affected than visual learning [16], [35]. Considering just one composite would not have been a perfect reflection of global cognitive function; it would have been misleading to assume that our patients were not suffering from other neuropsychological deficits [36]. It has been well documented in previous studies that it would be unusual to observe memory deficits in isolation [16], [37], and QoL measurement may be altered depending on the type of cognitive impairment [38]. Memory dysfunction is frequently associated with information-processing speed deficits or executive dysfunctions [39]. Our first study provided similar strong arguments to support the conclusion that MS patients with executive dysfunction are reliable and consistent when answering a self-reported QoL questionnaire (MusiQoL) [7]. It becomes necessary to report further information according to other definitions of cognitive dysfunction integrating combination of different composites.
5. How memory dysfunction is assessed should be discussed. We used the standardised free/cued recall test designed by Grober and Buschke [21]. Other tests could have been used. Memory assessment is also included in two well-validated neuropsychological batteries. In the Minimal Assessment of Cognitive Function in MS, auditory/verbal memory is explored using the California verbal learning test [40]. In the Brief Repeatable Battery of Neuropsychological Tests, verbal learning and memory is assessed using the selective reminding test [41]. The two main studies reporting similar data focused on memory assessment and defined memory dysfunction according to the Symbol Digit Modalities Test [6], the Wechsler Adult Intelligence Scale and the Wechsler Memory Scale (WMS-III) [5]. Our choice to implement the Grober and Buschke test relied on the following points: i) the ability to assess immediate and delayed memory; ii) the ability to assess free and cued memory; iii) the availability of a French version and French norms [21] that eliminated the need for a control group. Future studies should provide a synthetic index to categorize the patient as having impaired or non-impaired memory.
6. The confirmation of the metric properties in specific sub-populations is highly relevant; however, other elements should also be considered, such as content validity and acceptability. Future studies should explore, using factorial analysis, how the structure described in impaired samples fit with the initial structure of the tested instrument, which remains a key point when considering validity. The suitability indices to define satisfactory properties relied on arbitrary decision rules, each of which will be discussed. Nevertheless, this approach allows the homogenous determination of the suitability or unsuitability of different structures using the same decision tree. Future studies could test different decision trues and discuss what the results imply.
7. The analyses that we performed were designed to provide descriptive statistics and in particular to explore the external validity of comparisons between groups. We report the associations between the MusiQoL dimension scores and the sociodemographic and clinical features of our samples. These p-values were not used to draw conclusions but were rather used to reveal patterns. This is the reason we did not perform a correction for multiple comparisons.

### Conclusion

Our study confirms preliminary results reported in similar previous studies using different QoL measurements. These findings suggest that cognitive decline did not compromise the reliability or validity of self-reported health measures. If future studies provide similar results according to other definitions of cognitive dysfunction that integrate combinations of different composites (i.e., memory, attention, and concentration), assessment of QoL in MS patients could be more widely used without concern over the adequacy of this approach for cognitively impaired patients.

## Supporting Information

Table S1 **Psychometric properties of a quality of life questionnaire: definitions.**
(DOCX)

Table S2 **Internal structural validity/reliability/unidimensionality of the impaired and non-impaired short-delay free populations.**
(DOCX)

Table S3 **Internal structural validity/reliability/unidimensionality of the impaired and non-impaired short-delay total populations.**
(DOCX)

Table S4 **Internal structural validity/reliability/unidimensionality of the impaired and non-impaired long-delay free populations.**
(DOCX)

Table S5 **Internal structural validity/reliability/unidimensionality of the impaired and non-impaired long-delay total populations.**
(DOCX)
