# Supplementary material for: Relevance of Quality of Life Assessment for Multiple Sclerosis Patients with Memory Impairment
Source: PLoS One. 2012 Dec 11;7(12):e50056. doi: 10.1371/journal.pone.0050056 (PMC3519834; doi:10.1371/journal.pone.0050056)
Supplement: Table S1 — Psychometric properties of a quality of life questionnaire: definitions. (DOCX) [file pone.0050056.s001.docx]

**Table S1. Psychometric properties of a quality of life questionnaire: definitions**

| A valid QoL measure refers to the extent to which a concept is well-founded and corresponds accurately to the ‘real world’. The validity of a QoL measurement is considered to be the degree to which the tool measures what it claims to measure. Three main properties must be explored: reliability, internal validity and external validity.Reliability The reliability or internal consistency is the extent to which a measurement gives consistent results, i.e. the extent with which a set of items in a dimension measures the same attribute. Reliability is assessed by the computation of Cronbach’s alpha coefficients. Cronbach’s alpha coefficients higher than 0.70 result satisfactory reliability.  **Internal Validity**  Two main aspects must be considered: content validity and construct validity.   - Content validity is a non-statistical type of validity that involves the examination of the questionnaire content to determine whether it covers all the aspects of the domain to be measured. - Construct validity refers to the extent to which the questionnaire developed from a theory do actually measure what the theory says they do. It mainly relies on statistical analyses of the internal structure of the questionnaire including the relationships between responses to different items. Construct validity was assessed by performing: - Exploratory or confirmatory factorial analyses: in the case of confirmatory factorial analysis, a Kaiser-Meyer-Olkin (KMO) measure higher than 0.50 and a total variance higher than 70% indicate that the number of identified factors (or QoL dimensions) fit to the model; - Rash analysis to explore the unidimensionality of each domain identified: unidimensionality is retained if item goodness-of-fit (INFIT) statistics values range from 0.7 to 1.2; - Computation of correlation coefficients: correlation coefficients of each item with its dimension (item internal consistency, IIC) higher than 0.40 and higher than the correlation coefficients of this item with other dimensions (item discriminant validity, IDV) reflect a satisfactory construct validity.  External validity [External validity](http://en.wikipedia.org/wiki/External_validity) concerns the extent to which the internal construct can be support by external criteria. External validity relies on assessment of:   - Convergent validity: relationships between the dimensions of the questionnaire and the dimensions of other previously validated questionnaires measuring the same concept; - Criterion validity: relationships between the dimensions of the questionnaire and other features: sociodemographic, or clinical features… |
| --- |
