# Supplementary material for: Relevance of Quality of Life Assessment for Multiple Sclerosis Patients with Memory Impairment
Source: PLoS One. 2012 Dec 11;7(12):e50056. doi: 10.1371/journal.pone.0050056 (PMC3519834; doi:10.1371/journal.pone.0050056)
Supplement: Table S2 — Internal structural validity/reliability/unidimensionality of the impaired and non-impaired short-delay free populations. (DOCX) [file pone.0050056.s002.docx]

**Table S2. Internal structural validity / reliability / unidimensionality of the impaired and non-impaired short-delay free populations**

|  | M±SD | | IIC^1^ Min-Max | | IDV^2^ Min-Max | | Floor % | | Ceiling % | | Alpha^3^ | | INFIT^4^ | | Missing values % | |
| --- | --- | --- | --- | --- | --- | --- | --- | --- | --- | --- | --- | --- | --- | --- | --- | --- |
|  | NI 74 | I 38 | NI 74 | I 38 | NI 74 | I 38 | NI 74 | I 38 | NI 74 | I 38 | NI 74 | I 38 | NI 74 | I 38 | NI 74 | I 38 |
| ADL | 31,01±20,22 | 28,8±20,35 | **0,46**-0,69 | **0,38**-0,74 | -0,22-**0,47** | -0,4-**0,46** | 3 | 2,8 | 0 | 0 | 0,85 | 0,84 | 0,73-**1,65** | **0,63-1,64** | 1,9 | 4,9 |
| PWB | 52,71±24,14 | 48,44±24,48 | 0,54-0,83 | 0,61-0,81 | -0,06-0,43 | -0,3-0,61 | 0 | 5,6 | 1,5 | 0 | 0,86 | 0,81 | 0,72-1,34 | **0,59-**1,28 | 2,4 | 3,3 |
| RFr | 63,18±22,85 | 61,57±24,74 | 0,71-0,84 | 0,77-0,82 | -0,17-0,38 | -0,23-0,39 | 1,5 | 2,8 | 9 | 5,6 | 0,87 | 0,89 | **0,59**-1,18 | 0,92-1,05 | 1,8 | 2,6 |
| SPT | 57,09±22,56 | 49,83±25,2 | **0,38**-0,62 | **0,41**-0,66 | -0,21-**0,42** | -0,18-**0,43** | 0 | 0 | 3 | 2,8 | 0,72 | 0,75 | **0,67**-1,24 | 0,86-1,17 | 2,0 | 2,6 |
| RFa | 71,64±25,67 | 71,99±23,07 | 0,66-0,78 | 0,62-0,69 | -0,22-0,45 | -0,37-0,38 | 0 | 0 | 22,4 | 19,4 | 0,85 | 0,79 | 0,87-1,12 | 0,88-1,03 | 1,4 | 2,6 |
| RHCS | 68,66±19,41 | 69,91±19,95 | 0,51-0,67 | 0,45-0,57 | -0,08-0,41 | -0,15-0,23 | 0 | 0 | 10,4 | 8,3 | 0,73 | 0,68 | **0,68**-1,19 | 0,80-1,24 | 1,4 | 3,5 |
| SSL | 53,54±31,06 | 37,85±31,69 | 0,55-0,55 | 0,76-0,76 | -0,03-0,41 | -0,31-0,24 | 14,9 | 27,8 | 13,4 | 5,6 | 0,71 | 0,87 | 0,98-1 | 0,95-0,99 | 9,5 | 6,6 |
| COP | 55,22±27,28 | 54,86±31,09 | 0,41-0,41 | **0,53**-0,53 | 0,07-0,38 | -0,32-**0,56** | 4,5 | 8,3 | 10,4 | 8,3 | **0,58** | **0,69** | 0,99-1 | 0,91-0,99 | 1,4 | 2,6 |
| REJ | 63,43±32,3 | 73,26±31,79 | 0,8-0,8 | 0,77-0,77 | 0-0,4 | -0,24-0,65 | 6 | 5,6 | 26,9 | 44,4 | 0,89 | 0,87 | 0,97-0,97 | 0,91-0,94 | 1,4 | 2,6 |
| Index | 31,01±20,22 | 28,8±20,35 |  |  |  |  |  |  |  |  |  |  |  |  |  |  |

ADL activity of daily living, PWB psychological well-being, RFr relationships with friends, SPT symptoms, RFa relationships with family, RHCS relationships with health care system, SSL sentimental and sexual life, COP coping, REJ rejection

NI non-impaired, I impaired

^1^ Item-Internal Consistency, ^2^ Item Discriminant Validity, ^3^ Cronbach’s alpha, ^4^ Rasch statistics

Bold values: unsatisfactory values
