# Supplementary material for: Relevance of Quality of Life Assessment for Multiple Sclerosis Patients with Memory Impairment
Source: PLoS One. 2012 Dec 11;7(12):e50056. doi: 10.1371/journal.pone.0050056 (PMC3519834; doi:10.1371/journal.pone.0050056)
Supplement: Table S3 — Internal structural validity/reliability/unidimensionality of the impaired and non-impaired short-delay total populations. (DOCX) [file pone.0050056.s003.docx]

**Table S3. Internal structural validity / reliability / unidimensionality of the impaired and non-impaired short-delay total populations**

|  | M±SD | | IIC^1^ Min-Max | | IDV^2^ Min-Max | | Floor % | | Ceiling % | | Alpha^3^ | | INFIT^4^ | | Missing values % | |
| --- | --- | --- | --- | --- | --- | --- | --- | --- | --- | --- | --- | --- | --- | --- | --- | --- |
|  | NI 101 | I 21 | NI 101 | I 21 | NI 101 | I 21 | NI 101 | I 21 | NI 101 | I 21 | NI 101 | I 21 | NI 101 | I 21 | NI 101 | I 21 |
| ADL | 30±20,78 | 36,15±20,89 | 0,48-0,73 | **0,30**-0,83 | -0,24-0,44 | -0,39-**0,55** | 2,2 | 5 | 0 | 0 | 0,86 | 0,79 | 0,76-**1,59** | **0,4-1,63** | 2,8 | 6,0 |
| PWB | 48,17±24,97 | 62,5±20,58 | 0,59-0,83 | **0,40**-0,72 | -0,07-0,45 | -0,29-**0,55** | 3,3 | 0 | 1,1 | 0 | 0,86 | 0,69 | 0,73-1,15 | **0,55**-1,30 | 3,0 | 4,8 |
| RFr | 63,04±24,22 | 57,08±24,82 | 0,75-0,82 | 0,60-0,81 | -0,24-0,40 | -0,08-0,36 | 2,2 | 0 | 8,7 | 10 | 0,88 | 0,84 | 0,71-1,12 | 0,71-**1,40** | 2,3 | 4,8 |
| SPT | 54,96±22,88 | 54,69±28,45 | 0,36-0,61 | 0,61-0,78 | -0,15-0,35 | -0,26-0,46 | 1,1 | 0 | 2,2 | 10 | 0,70 | 0,86 | 0,72-1,20 | 0,77-1,20 | 2,5 | 4,8 |
| RFa | 72,55±24,97 | 66,67±24,03 | 0,64-0,72 | **0,48**-0,61 | -0,24-0,32 | -0,36-**0,60** | 0 | 0 | 21,7 | 15 | 0,82 | 0,71 | 0,92-1,10 | 0,84-0,98 | 2,0 | 4,8 |
| RHCS | 70,2±19,46 | 67,5±20,21 | 0,56-0,68 | **0,25**-0,52 | -0,11-0,27 | -0,25-**0,32** | 0 | 0 | 12 | 5 | 0,76 | **0,55** | 0,77-1,20 | **0,66**-1,21 | 2,0 | 6,3 |
| SSL | 49,73±32,36 | 40±30,78 | 0,62-0,62 | 0,63-0,63 | -0,07-0,30 | -0,53-0,57 | 18,5 | 25 | 12 | 5 | 0,76 | 0,77 | 1-1 | 0,86-0,99 | 8,9 | 7,1 |
| COP | 51,49±28,4 | 62,5±31,15 | **0,39**-0,39 | 0,72-0,72 | -0,06-**0,43** | -0,59-0,41 | 6,5 | 10 | 8,7 | 10 | **0,56** | 0,83 | 0,99-1.01 | 0,81-0,85 | 2,0 | 4,8 |
| REJ | 60,6±33,3 | 89,38±23,39 | 0,80-0,80 | **0,61**-0,61 | -0,04-0,45 | -0,47-**0,67** | 9,8 | 0 | 22,8 | 80 | 0,89 | 0,75 | 0,98-0,98 | 0,87-1,01 | 2,0 | 4,8 |
| Index | 30±20,78 | 36,15±20,89 |  |  |  |  |  |  |  |  |  |  |  |  |  |  |

ADL activity of daily living, PWB psychological well-being, RFr relationships with friends, SPT symptoms, RFa relationships with family, RHCS relationships with health care system, SSL sentimental and sexual life, COP coping, REJ rejection

NI non-impaired, I impaired

^1^ Item-Internal Consistency, ^2^ Item Discriminant Validity, ^3^ Cronbach’s alpha, ^4^ Rasch statistics

Bold values: unsatisfactory values
