# Supplementary material for: Relevance of Quality of Life Assessment for Multiple Sclerosis Patients with Memory Impairment
Source: PLoS One. 2012 Dec 11;7(12):e50056. doi: 10.1371/journal.pone.0050056 (PMC3519834; doi:10.1371/journal.pone.0050056)
Supplement: Table S4 — Internal structural validity/reliability/unidimensionality of the impaired and non-impaired long-delay free populations. (DOCX) [file pone.0050056.s004.docx]

**Table S4. Internal structural validity / reliability / unidimensionality of the impaired and non-impaired long-delay free populations**

|  | M±SD | | IIC^1^ Min-Max | | IDV^2^ Min-Max | | Floor % | | Ceiling % | | Alpha^3^ | | INFIT^4^ | | Missing values % | |
| --- | --- | --- | --- | --- | --- | --- | --- | --- | --- | --- | --- | --- | --- | --- | --- | --- |
|  | NI 66 | I 45 | NI 66 | I 45 | NI 66 | I 45 | NI 66 | I 45 | NI 66 | I 45 | NI 66 | I 45 | NI 66 | I 45 | NI 66 | I 45 |
| ADL | 32,01±20,79 | 27,83±19,52 | 0,46-0,71 | **0,34**-0,73 | 0-0,42 | 0-**0,53** | 3,3 | 2,4 | 0 | 0 | 0,86 | 0,82 | 0,73-**1,77** | **0,62**-**1,6** | 2,5 | 3,6 |
| PWB | 52,29±24,97 | 48,81±22,86 | 0,58-0,85 | **0,54**-0,77 | 0,01-0,50 | 0-**0,57** | 1,7 | 2,4 | 1,7 | 0 | 0,87 | 0,80 | 0,70-1,23 | **0,67**-1,29 | 2,7 | 2,8 |
| RFr | 63,75±22,07 | 60,91±25,67 | 0,73-0,83 | 0,76-0,8 | 0,01-0,34 | 0-0,41 | 1,7 | 2,4 | 6,7 | 9,5 | 0,88 | 0,89 | **0,63**-1,16 | 0,81-1,13 | 2,0 | 2,2 |
| SPT | 58,65±21,41 | 47,62±24,65 | **0,30**-0,57 | 0,46-0,66 | 0,02-**0,34** | 0,01-0,38 | 0 | 0 | 1,7 | 2,4 | **0,68** | 0,76 | 0,70-1,27 | 0,95-1,09 | 1,5 | 3,3 |
| RFa | 72,78±25,34 | 69,84±23,99 | 0,68-0,75 | 0,58-0,74 | 0,01-0,45 | 0,03-0,40 | 0 | 0 | 21,7 | 21,4 | 0,84 | 0,81 | 0,90-1,08 | 0,82-1,19 | 1,0 | 3,0 |
| RHCS | 70,14±18,11 | 68,85±20,17 | 0,44-0,61 | 0,48-0,61 | 0,02-0,33 | 0,02-0,33 | 0 | 0 | 11,7 | 7,1 | 0,69 | 0,71 | 0,71-1,26 | 0,81-1,21 | 1,5 | 3,0 |
| SSL | 54,79±29,03 | 36,39±34,3 | 0,48-0,48 | 0,81-0,81 | 0-0,38 | 0,03-0,34 | 8,3 | 35,7 | 13,3 | 7,1 | **0,65** | 0,90 | 0,98-0,99 | 0,95-0,99 | 9,1 | 7,8 |
| COP | 57,5±24,49 | 52,38±33,5 | **0,34**-0,34 | 0,54-0,54 | 0,02-**0,39** | 0,03-0,46 | 0 | 14,3 | 8,3 | 11,9 | **0,51** | 0,70 | 0,99-1 | 0,94-0,98 | 1,5 | 2,2 |
| REJ | 65,42±30,82 | 70,24±33,81 | 0,79-0,79 | 0,78-0,78 | 0,04-0,47 | 0,04-0,55 | 3,3 | 9,5 | 28,3 | 40,5 | 0,88 | 0,88 | 0,97-0,97 | 0,93-0,96 | 1,5 | 2,2 |
| Index | 32,01±20,79 | 27,83±19,52 |  |  |  |  |  |  |  |  |  |  |  |  |  |  |

ADL activity of daily living, PWB psychological well-being, RFr relationships with friends, SPT symptoms, RFa relationships with family, RHCS relationships with health care system, SSL sentimental and sexual life, COP coping, REJ rejection

NI non-impaired, I impaired

^1^ Item-Internal Consistency, ^2^ Item Discriminant Validity, ^3^ Cronbach’s alpha, ^4^ Rasch statistics

Bold values: unsatisfactory values
