# Supplementary material for: Relevance of Quality of Life Assessment for Multiple Sclerosis Patients with Memory Impairment
Source: PLoS One. 2012 Dec 11;7(12):e50056. doi: 10.1371/journal.pone.0050056 (PMC3519834; doi:10.1371/journal.pone.0050056)
Supplement: Table S5 — Internal structural validity/reliability/unidimensionality of the impaired and non-impaired long-delay total populations. (DOCX) [file pone.0050056.s005.docx]

**Table S5. Internal structural validity / reliability / unidimensionality of the impaired and non-impaired long-delay total populations**

|  | M±SD | | IIC^1^ Min-Max | | IDV^2^ Min-Max | | Floor % | | Ceiling % | | Alpha^3^ | | INFIT^4^ | | Missing values % | |
| --- | --- | --- | --- | --- | --- | --- | --- | --- | --- | --- | --- | --- | --- | --- | --- | --- |
|  | NI 102 | I 19 | NI 102 | I 19 | NI 102 | I 19 | NI 102 | I 19 | NI 102 | I 19 | NI 102 | I 19 | NI 102 | I 19 | NI 102 | I 19 |
| ADL | 29,72±19,49 | 39,67±26,57 | 0,43-0,71 | **0,46**-0,86 | 0,04-0,40 | 0-**0,65** | 2,1 | 5,9 | 0 | 0 | 0,82 | 0,91 | 0,72-**1,54** | **0,42-2,23** | 2,8 | 7,2 |
| PWB | 50,53±25,18 | 52,57±22,86 | 0,60-0,81 | **0,36**-0,88 | 0-0,49 | 0,01-**0,72** | 2,1 | 5,9 | 1,1 | 0 | 0,86 | 0,76 | 0,73-1,13 | **0,51-1,48** | 2,7 | 6,6 |
| RFr | 61,52±25,05 | 64,71±21,35 | 0,78-0,83 | **0,35**-0,73 | 0,03-0,40 | 0,02-**0,50** | 2,1 | 0 | 8,5 | 11,8 | 0,90 | 0,75 | 0,75-1,13 | **0,6-1,67** | 2,3 | 5,3 |
| SPT | 54,52±23,73 | 54,04±23,48 | 0,42-0,63 | **0,52**-0,6 | 0-0,29 | 0-**0,60** | 1,1 | 0 | 3,2 | 0 | 0,72 | 0,75 | 0,71-1,19 | 0,7-1,27 | 2,5 | 5,3 |
| RFa | 72,25±24,76 | 69,12±26,31 | 0,64-0,68 | **0,54**-0,75 | 0,01-0,33 | 0,01-**0,79** | 0 | 0 | 21,3 | 23,5 | 0,81 | 0,80 | 0,97-1,02 | 0,74-1,30 | 2,0 | 5,3 |
| RHCS | 70,21±19,19 | 71,08±18,89 | 0,52-0,69 | **0,24**-0,47 | 0,01-0,25 | 0-**0,43** | 0 | 0 | 11,7 | 5,9 | 0,75 | **0,53** | **0,68**-1,24 | 0,75-1,24 | 2,3 | 5,3 |
| SSL | 49,07±32,96 | 44,85±31,89 | 0,60-0,60 | 0,89-0,89 | 0-0,29 | 0,02-0,65 | 20,2 | 17,6 | 11,7 | 11,8 | 0,75 | 0,94 | 0,98-1,01 | 0,72-0,80 | 7,8 | **13,2** |
| COP | 52,39±28,24 | 59,56±33,81 | **0,43**-0,43 | 0,67-0,67 | 0,02-**0,49** | 0,01-0,63 | 6,4 | 11,8 | 7,4 | 17,6 | **0,60** | 0,79 | 0,99-0,99 | 0,82-0,86 | 2,0 | 5,3 |
| REJ | 63,83±33,37 | 80,88±30,97 | 0,81-0,81 | **0,71**-0,71 | 0,01-0,52 | 0-**0,83** | 8,5 | 5,9 | 28,7 | 64,7 | 0,89 | 0,83 | 0,98-0,98 | 0,73-0,99 | 2,0 | 5,3 |
| Index | 29,72±19,49 | 39,67±26,57 |  |  |  |  |  |  |  |  |  |  |  |  |  |  |

ADL activity of daily living, PWB psychological well-being, RFr relationships with friends, SPT symptoms, RFa relationships with family, RHCS relationships with health care system, SSL sentimental and sexual life, COP coping, REJ rejection

NI non-impaired, I impaired

^1^ Item-Internal Consistency, ^2^ Item Discriminant Validity, ^3^ Cronbach’s alpha, ^4^ Rasch statistics

Bold values: unsatisfactory values
